# The educational integration of digital technologies preCovid-19: Lessons for teacher education

**DOI:** 10.1371/journal.pone.0256283

**Published:** 2021-08-19

**Authors:** Jesús Valverde-Berrocoso, María Rosa Fernández-Sánchez, Francisco Ignacio Revuelta Dominguez, María José Sosa-Díaz

**Affiliations:** Department of Educational Sciences, Teacher Training College, University of Extremadura, Cáceres, Spain; University of South Australia, AUSTRALIA

## Abstract

The educational integration of Information and Communication Technologies (ICT) has been put to the test because of the need to implement «emergency remote education» as a result of COVID-19. Within this context of uncertainty («viral modernity»), flexible education is an option to promote a more just, equitable, accessible and creative educational system. In order to properly interpret the effects of this unique educational circumstance, it is essential to study the previous situation in terms of the use of digital technologies in teaching practices. The objective of the study is to describe the educational integration of ICT and the teacher education model to obtain evidence that contributes to understanding the phenomenon. To this end, a questionnaire consisting of two self-reporting tools and a scale on the description of teaching practice with ICT was applied. The sample is made up of teachers from public primary and secondary schools (N = 251). Data collection was carried out in the months prior to the closure of schools due to the Covid-19 pandemic. A univariate analysis of the variables and contrast tests of non-parametric hypotheses was carried out, along with calculation of the reliability and construction validity of the measuring instruments. The results reveal the most frequent types of teaching practice with ICT and the spaces where digital technologies are commonly used. Various weaknesses can be identified in digital competence among teachers, as well as in the initial/continuing training model, which contribute to the understanding of the difficulties encountered during "emergency remote education". Participation in ICT didactic innovation projects and the performance of ICT Coordination are associated with more experiential training. Flexible education requires a redefinition of the teacher training model that encourages learning anywhere, anytime.

## Introduction

The current pandemic is forcing education systems to face many challenges that raise very important questions about the future of education. Teachers have been forced to implement, during the closing of schools, an "emergency remote education", very different from planned practices such as distance education, e-learning or b-learning, with very mixed results and the revelation of weaknesses in the system such as the digital divide, inequity or social injustice.

The concept of "Global Pedagogical Blackout" refers to the transition between the Third and Fourth Industrial Revolution, through which [1] has occurred: (A) a progressive despedagogization of educational reality; (b) the construction of an evaluative culture based on a restrictive concept of quality and relevance; (c) the development of a model of thought based on the "crisis" of the education system; (d) a significant decrease in investment in education, especially in the updating of digital technologies; (e) the application of an outdated curriculum; and (f) the gradual conversion of the teaching profession into a mere curriculum manager.

To try to make sense of the Covid-19 pandemic, one must consider the complex interaction of "viral behaviors" in all spheres of life. Hence the new concept of "viral modernity", an example of "bio-informationalism", which applies to "viral technologies, codes and ecosystems in information systems, publication, education and emerging knowledge" [2]. In this unstable environment, Veletsianos & Houlden [3] maintain the need for a flexible education (teaching and learning anywhere, anytime) that promotes a more just, accessible, autonomous, and creative system. Flexible education, so that it does not become a discriminatory option, must be compatible with socio-constructivist pedagogical approaches such as cooperative learning, learning communities, networked learning or peer learning, supporting students by considering their individual characteristics and the context in which they are developed [4].

The use of digital technologies in classrooms is still far from generating systemic change, rather promoting “islands of innovation”, based on the work of excellent teachers who carry out innovation in their teaching practices using Information and Communication Technologies (ICT) without mediating a formal process of lifelong learning [5]. In fact, some contextual variables such as the school climate or trust within the educational center, the role of the ICT coordinator and the management team, as well as the existence of networks for access to new information and knowledge sharing among teachers, have a greater positive effect on the use of ICTs than traditional lifelong learning activities [6–9].

Teacher training must go beyond the development of basic digital skills and seek strategies to integrate the interpretative and creative potential of ICT into their training actions. Most teaching practices make "superficial" or "basic" use of digital technologies for pre-class preparation, personal communication, use of word processing, slide presentations or search for information. Teachers feel that they are not sufficiently prepared to use ICT and that the incorporation of new methodological approaches is not sufficiently encouraged. Røkenes & Krumsvik [10] identified, from a literature review, a series of approaches to teacher training in educational technology and subsequently studied their effectiveness in a specific training program, with the following typologies [11]:

- Collaborative approach: Development of digital competence among teachers through knowledge-building technologies, both synchronous and asynchronous, which include online forums, video conferencing systems, use of social networks and web 2.0 tools (online collaborative documents), blogs or specific software for collaborative learning. These activities also enable collaboration to be enriched with the participation of others (students, teachers, trainers, specialists, researchers) from different geographical contexts. These training proposals enable the development of team work competencies.
- Metacognitive approach: In this form of training the teacher is directed to carry out a reflection on the didactic action by means of an expression and analysis of his thoughts, behaviors and/or consequences of his professional practices in any situation where digital technologies are involved. Training experiences that use this approach implement, for example, online communication tools, case videos, or microteaching to stimulate critical assessment of ICT use in classrooms. The results of the research show the need for teachers who are in training to gain in-depth knowledge of theories, to justify and understand their own practice and professional decisions, related to the integration of digital technologies into the curriculum.
  Although there is an over assessment of practical ICT activity to develop digital competence, teachers also demand theoretical training to enable them to develop arguments that justify the use of technologies in their teaching practice.
- Mixed learning approach: Uses a combination of classroom and non-classroom training to develop digital teaching skills through learning experiences with a variety of technological resources with which one can interact and carry out learning activities.
  The modality itself directly trains teachers in the use of online learning platforms, as well as in the use of digital materials for training purposes.
  This modality is particularly appropriate for the teacher’s development as a reflective professional because, by increasing the channels available for communication, it is possible to establish debates that foster a critical attitude toward the use of ICT in teaching and adopt a position with regard to ethical problems such as cyberbullying and plagiarism.
- Modelling-based approach: the trainers act as tutors or mentors to promote certain practices or to offer different visions of the teaching-learning process, through the intentional demonstration of specific action guidelines or teaching strategies related to the integration of digital technologies that can have real impact in the classroom. The hypothesis is that when the trainer shares relevant experiences, examples and strategies on the educational use of ICTs through pedagogical reasoning based on clear, explicit and understandable teaching practice, the digital competence of the educator in training is promoted. Research results show that using strategies, such as examples of how to perform a task with ICT while expressing the mental process aloud, or direct demonstration of how to use a digital resource didactically, have positive effects on digital teaching skills. However, some weaknesses are identified, such as: (a) the passive role of the teacher in training before modelling, without possibility for action; (b) that the "model" is not perceived by the teacher in training with the expected level of digital competence; and (c) the adoption by "mimetism" of certain ICT-modelled practices without adopting a critical, reflective and context-sensitive position.
- Authentic learning approach: Learning activities represent real-world situations pertaining to the educational context close to the teacher in training. This modality involves teachers in the design, elaboration, application and evaluation of real teaching practices with technologies. They may involve the specific creation of teaching materials and are carried out with the guidance of specialized trainers. It is a formative experience that allows the building of bridges between theory and practice, immerses the teacher in training in active and meaningful learning with positive effects on the perception of their self-efficacy with technologies in the classroom. Research has shown that the most useful form of training is that in which leaders use ICT in their own practice and offer opportunities for teachers in training, beyond a "demonstration". Teachers demand significant learning experiences with ICT because they do not consider themselves able to perceive the real possibilities of digital technologies in the teaching-learning process and therefore do not appropriate ICT for their classes. These results underscore the importance of providing real-world practice opportunities for the development of digital teaching skills and their impact on the development of positive attitudes and self-efficacy perceptions. In cases, the following factors are emerging as essential to teacher training for the curricular integration of digital technologies: (1) The commitment to develop a shared vision of the role of ICT in the educational process and (2) the participation of teachers in communities of practice or professional networks. On the other hand, it is considered necessary to bridge the gap between research and educational practice. Thus, the main challenges facing teacher training are [12]: The contextualization of their actions to the socio-cultural, economic, historical and political reality of the specific environment [13]; the development of sustainable and scalable training, through the promotion of communities of practice or online professional networks [14]; didactic innovation with digital technologies in teacher training actions, which model future teaching practices in their classrooms [15]; inclusion in teacher training of ICT decision-making strategies in evidence-based education (products, services or processes), knowledge of context and ethical values [16].

In March 2020, we were closing schools because of the COVID-19 pandemic. At that time, the relationships between students and teachers began to be mediated, in their entirety, by technological tools, and the so-called "emergency remote education" was developed [17, 18]. Educational centers were definitively placed in a virtual space as one of the gears in the educational network, establishing themselves as virtual environments of training and knowledge production. International agencies are looking for ways to guide a clear educational response to the COVID-19 pandemic by urging them to compensate for holes in student access to technology, with special emphasis on students in disadvantaged situations, to increase the training and digital competence among faculty members, in order to articulate systems of platforms and digital resources for the online communication between students andAnalysis of the digitization process of a Secondary School from the DigCompOrg model teachers, as well as offering a variety of open digital teaching materials for completing school work from home. The OECD [19] is conducting a study that includes 98 countries, whose report reveals some of the priority considerations and responses that education systems have for the so-called "new normal". Identified challenges include: Ensuring continuity of academic learning for students, supporting those lacking independent study skills; ensuring continuity and integrity of learning assessment; ensuring family support so they can guide learning; ensure the well-being of students and faculty. The main barriers they have encountered have been: Availability of technological infrastructure, addressing the emotional well-being of students, addressing the right balance between digital activities and tasks without screens, and managing technology infrastructure. UNESCO has also published its own documents analyzing the impact of the pandemic on school systems, providing recommendations to managers and government administrations for the reopening of schools [20–22]. In the field of educational research, studies have been published in recent months on the pedagogical response to the total or partial closure of educational centers, with approaches to rapid solutions, especially aimed at teachers. This raises doubts within the educational community about the quality of the teaching that has been developed [23, 24]. Spoel et al. [25] found that average prior experience in the educational use of digital technologies influenced a more positive perception of emergency remote education than expected during the school closure due to the pandemic. Neither teachers without experience or those with extensive experience had their initial expectations altered. Fernández & Prendes [26] consider that the digital transformation of schools should be consolidated by evaluating a digital education plan that encourages critical reflection on the role of technologies in learning. Teachers demand better technical support, a higher quality of open educational resources and an update of technological devices. Fernández-Batanero et al. [27] in a systematic review of the literature, found that there are few studies on the development of digital teacher competence and most conclude that teacher training is insufficient, despite being considered a key factor in improving professional performance. Pre-pandemic studies have been lacking in relation to the starting situation, teaching practices and the training of teachers in educational technology that would allow for a pedagogically adequate response. From this point of view, we wondered whether the teachers were prepared to move from classroom to online teaching. We analysed what common teaching practices with digital technologies were teachers applying just before the school closed. We questioned the approach used in initial/ongoing teacher training prior to the pandemic. We wondered about the relationships existing between initial/ongoing teacher training and participation in IT teacher innovation projects and the performance of ICT Coordination positions. With these research questions in mind, the objectives (O) of our study are:

O1. Describe common teaching practices with digital technologies and digital teaching competence for the educational integration of ICTs before the pandemic.
O2. Determine the approach used in initial/permanent teacher training prior to the closure of schools due to the pandemic.
O3. To analyze the relationships that exist between characteristics of ICT teaching received by staff and the following variables: (A) participation in ICT didactic innovation projects and (b) performance of the post of ICT Coordinator.

## Materials and methods

### Design

It is a survey-type investigation in which the processing of information is descriptive and comparative in nature. This study is part of a research project whose aim is to design an Integrated Digital Education Plan (PIED) in primary and secondary schools for the improvement of learning outcomes, oriented under the principles of autonomy, contextualization, efficiency-effectiveness and didactic innovation. A central focus of this research is the redefinition of the teacher training model, which requires an investigation into teachers’ perception of their digital competence, identification and categorization of classroom ICT educational practices and knowledge of whether the perspective used in prior training (initial or permanent) is compatible with the most innovative approaches.

The research process has been divided into four distinct phases (**S1 Fig**): (A) Problem approach, in which the reference bibliography has been consulted mainly; (b) Research design in which the research instruments were selected; c) Field work in which the online questionnaires were applied; and finally d) The analysis of data, taking into account that the application of the questionnaires was immediately prior to the confinement by COVID-19 and the development of "remote emergency education" and involved a redefinition of the initial problem and the final conclusions.

### Participant

The sample was selected from the set of public primary and secondary education centers in Extremadura (Spain), which have a Digital Education Plan (Instruction 20/2018 of the General Secretariat of Education of the Ministry of Education and Employment of the Board of Extremadura, Spain). It is composed of a total of 251 primary (40.6%) and secondary (59.4%) teachers in public centers. The majority are women (64.1%) and civil servants (69.7%). The average age of teachers is 45 years (DT = 8.73). Three subgroups, with a similar percentage representation, are identified with respect to years of professional experience: 10 years or less (33.9%), 11 to 20 years (35.5%) and over 20 years (30.3%). The total percentage of teachers in the sample who occupy (or have occupied) the position of ICT coordinator in their educational center is 14.3%. More than half of the sample faculty (53.4%) have participated in one or more ICT-related educational innovation projects.

### Instruments

An online questionnaire with three components was applied [28]: Two self-report scales (SQD-Scale and TICTIP Scale) that were translated into Spanish, and an additional series of 22 items based on the Learning Design Support Environment project [29]. The questionnaire was designed to promote the use of digital technologies in the teaching-learning process. These items were organized around three dimensions: (a) spaces used for teaching-learning with ICT (8 items), (b) learning outcomes expected from the use of ICTs (8 items), and (c) type of teaching practice performed with ICT (6 items). The latter dimension is based on the learning typologies of the "Conversational Framework" [30]. A Likert-type scale of six elements was used: (1) Never, (2) Almost Never, (3) Sometimes, (4) Often, (5) Very often, and (6) Always. Lastly, a number of items of teacher data were included. SQD-Scale (Synthesis of Qualitative Evidence) is a self-reporting tool based on a theoretical model called SQD-Model [31], which measures teachers’ perceptions of the degree to which they experience the support and training needed to integrate digital technologies into their teaching practice. It consists of 24 items, grouped into six dimensions [32]. Cronbach’s Alpha (α = .95) and McDonald’s Omega (ω = .95) were used as estimates for the reliability of the entire SQD scale. The latter is considered to be more robust and suitable for measuring instruments that are generally applied in the Social Sciences [33]. SQD uses a six-element Likert scale: (1) Strongly disagree, (2) Disagree, (3) Slightly disagree, (4) Slightly agree, (5) Agree, and (6) Fully agree. Each of the 6 SQD dimensions showed the following reliability:

Use of teacher trainers for modelling (α = .88; ω = .88), Reflection on attitudes toward the role of technology in education (α = .85; ω = .85), Instructional design with technologies (α = .88; ω = .86), Collaboration with other teachers (α = .82; ω = .82), Scaffolding on authentic experiences (α = .80; ω = .81), and Changing from traditional assessment to continuous feedback (α = .86; ω = .87). These values are similar to those reported by the authors of the scale [20]. An exploratory factorial analysis (AFE) was carried out to verify the validity of the Spanish version of the construction, and it was verified that it was added to the SQD model of six components. The «Scale for teachers’ ICT integration proficiency (ICTTIP)» is a self-reporting tool that measures teacher skills related to the educational use of digital technologies [34]. It is structured in six subscales: Information collection and preparation (2 items), Materials production and problem solving (5 items), Communication and sharing (2 items), Planning, teaching and evaluation (10 items), Teacher training and self-learning (3 items) and Ethics, Health and Safety (5 items). A Likert scale of six elements is used: (1) Never, (2) Almost Never, (3) Sometimes, (4) Often, (5) Very often, and (6) Always. The ICTTIP has shown a high reliability coefficient (α = .95; ω = .95). The calculation of the responsibilities for each of the subscales of the questionnaire shows the following coefficients: Preparation (α = .84; ω = .85), Production (α = .82; ω = .82), Communication (α = .70; 2 items), Teaching (α = .92; ω = .92), Training (α = .82; ω = .82), Ethics, Health and Safety (α = .80; ω = .81). Exploratory factor analysis (AFE) for the construct validity of the Spanish version is compatible with the six-component structure of the ICTTIP.

### Data analysis

Descriptive statistics (measures of central tendency and variability) have been calculated for exploratory analysis of the data. These analyses are completed by the calculation of Kolmogorov-Smirnov normality tests for each of the research hypotheses that are compared. For the confirmatory analysis of the data, a univariate analysis of each variable (relative and absolute frequencies, central trend statistics and dispersion) and non-parametric hypothesis contrast tests (Mann-Whitney U) have been used. Rosenthal r was used to measure the size of the effect. For the measuring instruments: their reliability (Cronbach’s Alpha and McDonald’s Omega) and construct validity (Exploratory Factor Analysis) have been calculated. The questionnaire data was collected between 30 January and 3 March 2020 [35]. The data analyses were conducted using SPSS 25 and JASP 0.14.1.

### Ethics

This study was approved by the Ethics Committee of the University of Extremadura (Spain). We conducted this study in accordance with the Ethical guidelines for educational research [36] from the British Educational Research Association (BERA). All participants agreed to participate voluntarily, with informed consent when they fill the survey. and were able to withdraw from the study freely at any time. All questionnaires were designed and applied to ensure anonymity of participants. The data was confidential and participation was anonymous without any potential risk to the integrity of the subjects.

## Results

### Description of results on common ICT teaching practice and digital teaching competence

In response to the first research objective, several items of the instrument based on the Learning Design Support Environment project and the "Scale of Teaching Competence for ICT Integration" (TICTIP) are analyzed.

First, the educational contexts in which technologies are used are analysed including the typology of teaching practices and the expected learning outcomes with the use of ICT.

#### Educational features used with ICT

The teachers in the sample mostly use the classroom as the educational space for integrating digital technologies, with the highest average frequency of use (Table 1). In second place, is the family physical context (home) which is most often used for the educational use of ICTs. Within the spaces of the educational center, the next most commonly used spaces are the IT classrooms or computer classrooms, followed by the library. In both cases the average frequency of use is low. In other areas of schools such as laboratory classrooms or those specific to physical education activities, the use of digital technologies is very rare. It is observed that there is moderate use of the virtual classroom, understood as the use of Learning Management Systems (LMS) platforms (e.g. Moodle), classroom management (ClassDojo) or other resources of web 2.0 (e.g. blogs). The b-learning modality is rare, as reflected by the results of the use of the «inverted classroom» or «flipped-classroom».

**Table 1:** Description of the usual teaching practices with ICT: Spaces, learning results and typology.

**Spaces used for ICT teaching-learning**	**M**	**DT**
Generic classroom	5.04	1.139
Technology classroom (IT room or Computer room)	2.71	1.675
Laboratory classroom	1.78	1.331
Physical Education classroom	1.38	.958
Library	2.57	1.546
Student’s personal space (e.g. the home)	3.49	1.381
Flipped Classroom (classroom + student’s personal space)	2.33	1.509
Virtual classroom (e.g. ClassDojo. Moodle. Blogs. …)	3.03	1.760
**Learning results hoped to achieve with the use of ICT**	**M**	**DT**
Knowledge (define, identify, remember, list. . .)	4.32	1.187
Understanding (classify, explain, ask, select. . .)	4.51	1.154
Application (demonstrate, find, predict, build. . .)	4.49	1.118
Analysis (differentiate, relate, compare. . .)	4.45	1.160
Synthesis (generalize, combine, conclude, explain reasons for. . .)	4.30	1.215
Evaluation (criticize, give arguments for and against, judge, reflect. . .)	4.19	1.279
Attitudinal (show awareness towards, be receptive to, value. . .)	4.33	1.267
Psychomotor (do, perform, draw, develop a physical exercise. . .)	3.25	1.486
**Type of teaching practice done with ICT**	**M**	**DT**
Read/View/Listen (Exhibit)	4.78	1.105
Collaborate (cooperative)	4.37	1.256
Debate-Reflect (communicative)	4.13	1.333
Investigate (indagative)	4.75	1.181
Practice (application)	4.65	1.182
Produce (creative)	4.30	1.360

#### Typologies of teaching practices

Teachers use digital technologies more frequently as a resource to support expositional teaching practices (read/view/listen). Secondly, the most frequent activities of ICT use are those involving research, that is, conducting research or exploration tasks. Thirdly, teaching practices that guide the student’s tasks toward the application of knowledge are put in place. Teaching methodologies enriched with less common technologies are: (a) collaborative activities involving the development of cooperative learning; (b) learning tasks aimed at the creation or production of digital resources by students (e.g. texts, images, audiovisual, etc.); and (c) ICT-supported communication activities involving debate and reflection. Globally, it is observed that teachers use all the practices identified in the questionnaire with a similar frequency, which is at a medium-high level, according to their perception.

#### Expected learning outcomes

The most frequent learning outcomes that teachers achieve with the use of ICTs are first to be seen with regards to «understanding», that is student achievement involving competencies for classification, explanation, or question formulation. Upon close assessment, «application» is established, i.e. the achievement of learning outcomes linked to demonstration, prediction or elaboration. Thirdly, «analysis» or ability to differentiate, relate and compare is identified as an achievement made through ICT. At the same time, three less frequent learning outcomes obtained through digital technologies are pinpointed: (a) «synthesis», or ability to generalise, combine or conclude; (b) «evaluation», which involves the ability to critique, to argue or make judgments; and (c) achievements in the «psychomotor» field, which have to do with results in physical or manual activities. In intermediate positions, there is an indication of learning results related to the development of attitudes (awareness, receptivity) and the acquisition of knowledge, i.e. to be able to define, identify, remember or list.

With regards to the digital teaching competence for the integration of digital technologies, the "Scale of Teaching Competence for the Integration of ICT" (TICTIP) is analyzed, offering the results by dimensions.

### Descriptive results from the «Scale on Teaching Competence for ICT integration» (ICTTIP)

#### Dimension 1: Preparation

Teachers demonstrate the very frequent use of the Internet to search for information that is subsequently provided to students as a complementary educational resource (Table 2). They also use the computer for the development of classroom resources, for gathering teaching materials and for the evaluation of activities at very high frequency. They also spend time, on a daily basis, on the selection of ICT resources suitable for the development of the school curriculum.

**Table 2:** Description of the usual teaching practice with ICT: Spaces, learning results and typology (ICTTIP).

Regular teaching practice with ICT	M	DT
1. I use the computer to develop classroom resources, teaching materials and evaluation activities.	5.06	1.045
2. I use the Internet or other information technology, to search for information that I provide to students as a complementary educational resource.	5.31	.809
3. I spend time selecting ICT media or resources that suit the curriculum.	4.90	1.084
4. I use presentation software for my exhibition classes.	4.13	1.353
5. I am able to solve technical problems during class (e.g. when the projector/digital whiteboard does not recognize the computer).	4.01	1.301
6. I apply the objectives, content and evaluation criteria related to the development of digital competence set out in the curriculum.	4.29	1.169
7. I use my computer to record or edit sounds/music as teaching material.	3.88	1.538
8. I use ICTs to introduce new teaching methodologies.	4.43	1.254
9. I use email, instant messaging (e.g. Scratch) or the web to communicate with my students.	4.31	1.689
10. I use blog/web to share knowledge or answer questions posed by students.	3.25	1.783
11. I teach students how to find useful web resources for academic learning.	4.29	1.249
12. In classes where I use ICT, I divide students into groups.	3.56	1.338
13. I make sure that all students have enough ICT resources and skills to perform their academic tasks.	4.25	1.252
14. I provide worksheets to students when I ask them to use web information to complete their homework.	3.58	1.474
15. I use ICTs to foster students’ high-level thinking capacity, such as creativity, analysis, and judgment.	4.05	1.287
16. I evaluate students’ digital competence as a complement to academic qualification based on written tests (exams).	3.34	1.473
17. I value and rate student learning progress when participating in ICT-supported group activities.	3.75	1.399
18. I evaluate my teaching practices with ICT to improve my classes.	3.75	1.431
19. I design academic tasks with ICT integration so that students without a computer at home can also participate.	3.65	1.604
20. I design different ICT learning activities for students with different levels of performance.	3.57	1.436
21. I spend time learning and practicing ICT skills.	4.30	1.157
22. I use courses and other online materials for my professional training.	4.53	1.187
23. I have attended conferences/conventions or read specialized journals to learn about methods for ICT integration.	3.71	1.439
24. I teach students about ethical rules and norms with regards to the Internet before students use it.	4.23	1.417
25. I demand that students respect intellectual property rights.	4.35	1.446
26. I am aware of problems among teens with Internet addiction and access to adult content pages.	5.41	.878
27. I tell students how abusive use of digital devices can affect their health.	5.15	1.069
28. I follow-up with students who demonstrate low motivation and academic performance due to Internet addiction.	3.41	1.581

#### Dimension 2: Production

The use of ICTs often favors the introduction of new teaching methodologies, as well as the application of objectives, content and evaluation criteria related to the development of the digital competence established by the curriculum. The use of presentation software for exhibitive classes is quite common. There is a capacity to solve technical problems during class development in most cases. The use of technologies for the creation or editing of sound material for teaching purposes (podcast, music) is rare.

#### Dimension 3: Communication

Teachers often use email, instant messaging or the web for communication with students, especially through specific communication and educational management platforms provided by the administration (v.gr. Rayuela). However, the use of blogs for knowledge sharing or student tutoring is a little-used resource.

#### Dimension 4: Teaching (planning, teaching and evaluation)

Faculty often teaches students how to conduct web searches of educational resources useful for their academic progress. Furthermore, in general, teachers find that all their students possess sufficient ICT resources and skills for the performance of their school activities. Teachers often use ICTs to foster high-level thinking skills such as creativity, analysis, and judgment. Evaluation of learning outcomes in group activities, with ICT support, is used in student scoring infrequently. Similarly, it is rare to evaluate ICT teaching itself, with the aim of improving teaching. Every now and then, teachers design academic tasks with ICT so that students who do not own a computer at home can participate. The least frequent teaching activities are: (a) the assessment of students’ digital competence and their complementary consideration in relation to learning outcomes on written tests (tests); (b) the division into groups of students in a classroom when using ICT; (c) design of specific learning activities for students with different levels of performance; and (d) providing students with job cards/guides for managing web information in performing school duties.

#### Dimension 5: Training (professional development and self-learning)

Faculty often use online courses and materials for ongoing training. In addition, one often spends time learning and practicing competencies related to digital technologies. However, participation in professional meetings (congresses, conferences) or reading articles in scientific journals on the educational integration of ICTs is rare.

#### Dimension 6: Ethics, health and safety

Teachers often express awareness of the problems of addictive use of the Internet and access to inappropriate content among adolescents. Teachers often communicate to their students regarding health problems that can arise from misuse of digital devices. The demand for students to respect intellectual property rights is quite common. Teachers admit that they often teach ethical standards and rules about the use of the Internet to students in a preventive manner. However, it is unusual for teachers to follow-up with students who show low motivation and academic performance due to excessive reliance on digital devices.

### Approach used in preservice/inservice teacher training prior to school closure

In order to respond to the second objective, we analyzed the results by dimensions of the SQD Scale, which inform us of the approach used in the preservice/inservice training of the surveyed teachers.

#### Dimension 1: Modelling

Teachers say that they know of some examples of using ICTs in educational contexts, but that they cannot classify this knowledge as very extensive in a number of cases (Table 3). In the same sense, they claim that they have been able to observe some good ICT educational practices that have inspired them to application in their classrooms, recognizing that their impact on teaching practice is limited. Teachers maintain that they have had some experience in the use of ICT in educational contexts but recognize it as insufficient to be able to integrate these technological resources into professional practice by themselves. Moreover, they consider that concrete demonstrations of the potential for the use of ICTs in education have been limited.

**Table 3:** Description of the initial and/or permanent teaching training experience (SQD scale).

During my initial and/or permanent teaching training. . .	M	DT
1. I have seen many examples of ICT use in educational contexts.	4.27	1.166
2. I have observed sufficient ICT use in educational contexts to be able to integrate these technological resources into my professional practice myself.	3.94	1.187
3. I have seen examples of good ICT educational practices that have inspired me to apply them in my classrooms.	4.12	1.294
4. I have received concrete demonstrations of the potential for ICT use in education.	3.83	1.361
5. I have had the opportunity to reflect on the role of ICT in education.	4.28	1.170
6. I have discussed the challenges of integrating ICTs into education.	3.96	1.213
7. I have been offered the opportunity to discuss my own experience with ICT in the classroom.	3.26	1.371
8. There were specific occasions when the general attitude towards ICT in education was debated.	3.49	1.306
9. I have received sufficient advice in the design of ICT-enriched learning activities.	3.34	1.327
10. I have learned how to integrate ICT into my teaching practice in the classroom.	4.00	1.180
11. I have received technical and pedagogical support for the production of teaching materials.	3.25	1.341
12. I have gained a lot of support to develop ICT-enriched educational activities and projects.	3.16	1.284
13. I have had quite a few occasions when I have worked with other colleagues on the use of ICT in education (e.g. projects of didactic innovation, realization of educational materials, etc.).	3.23	1.297
14. I have become convinced of the importance of cooperation in the use of ICTs in education.	4.47	1.288
15. Teachers helped each other to use ICTs in educational contexts.	3.90	1.311
16. I have shared teaching experiences on the use of ICTs.	3.67	1.397
17. There were quite a few occasions when I was able to consider different ways to use ICT in the classroom.	3.56	1.190
18. I have been able to learn how to use ICT in classrooms through internships with trainers.	3.42	1.329
19. I was motivated to gain experience in the use of ICT in classrooms.	3.69	1.351
20. Teachers themselves were motivated by each other when they tried to use ICT in an educational context.	3.89	1.225
21. I have received sufficient advice on the use of ICTs in my teaching practice.	3.29	1.277
22. My ICT skills have been thoroughly assessed.	2.70	1.174
23. I have obtained sufficient feedback on how to develop my competence in ICT use in the future.	2.97	1.117
24. My competencies in ICT use in the classroom were regularly evaluated.	2.56	1.120

#### Dimension 2: Reflection

Teachers recognize that they have had some opportunity to reflect on the role of ICT in education. One has occasionally been able to discuss the challenges of integration but one has hardly been given opportunities to discuss the general attitude towards ICT in education or one’s own experience with ICT in the classroom.

#### Dimension 3: Instructional design

Teachers agree somewhat that they have learned how to integrate ICT into their teaching practice in the classroom. However, they feel that they have not received sufficient advice in the design of ICT-enriched learning activities. Nor are they satisfied with the technical and pedagogical support received for the elaboration of didactic materials. In this way, teachers believe that the support received to develop ICT-enriched educational activities and projects has not been sufficient.

#### Dimension 4: Collaboration

Teachers believe that cooperation with regard to the use of ICTs in education is of some relevance but does not become a prominent variable. They have not observed that teachers routinely assist each other in the use of ICTs in educational contexts. Teachers believe that there have not been enough occasions when they have worked together with other teachers on the use of ICT in education, such as didactic innovation projects or the realization of educational materials.

#### Dimension 5: Authentic experiences

Teachers have not observed that they are sufficiently motivated among themselves when they try to use digital technologies in the educational context. Individually, they have not received the necessary stimulus to gain experience in the use of ICT in the classroom. Teachers believe that they have not had enough opportunities to assess different uses of ICT in the classroom and that the results of their training for the use of technologies in the classroom, through practices tutored by advisors, do not reach the desired quality.

#### Dimension 6: Feedback

Teachers believe that the advice received on the use of ICT in their teaching practice has been scarce. They argue that they have not obtained enough feedback on how to develop their digital teaching skills in the future. Teachers state that their competencies on the use of ICTs have not been evaluated in depth, nor on a regular basis.

### Analysis of the relationships between participation in ICT innovation teaching projects and SQD

First, we respond to the first variable analyzed in relation to the third objective. In this case we analyze the relationships that exist between the characteristics of ICT teaching among staff and the participation in projects of didactic innovation with ICT, obtained from the SQD Scale.

Statistically significant differences in participation/non-participation in ICT innovation projects have been observed in all SQD variables (Table 4). (1) In the "Modelling" dimension with: (a) Observe many examples of ICT use in educational contexts. (b) To get to know the educational uses of ICTs in order to carry out, in an autonomous way, the integration of these resources into teaching practice. (c) Observe good educational practices with ICT that inspire their application in the classroom and (d) Receive specific demonstrations of ICT’s educational potential. (2) In the "reflection" dimension with: (a) Thinking about the role of digital technologies in education. (b) Discuss the challenges of ICT curriculum integration. (c) Discuss the personal experience of using ICT in the classroom and (d) Discuss attitudes toward the use of digital technologies in educational contexts. (3) In the "Instructional Design" dimension with: (a) To receive advice for the design of ICT learning activities. (b) Learn how to integrate ICT into teaching practice. (c) To receive technical and pedagogical support for the realization of didactic resources and (d) Obtain support to develop ICT-enriched educational activities and projects. (4) In the "collaboration" dimension with: (a) Working in teams on the educational use of ICTs. (b) To assess the relevance of cooperation to the use of digital technologies. (c) Have collaborative experiences among colleagues to integrate ICTs and (d) Share experiences with other teachers. (5) In the "authentic experiences" dimension with: (a) To evaluate different uses of ICT in the classroom. (b) Learning to use ICT through practices with specialized trainers. (c) Be motivated to acquire teaching experiences with ICT and (d) Experience mutual motivation toward ICT among teachers. Finally, (6) In the "Feedback" dimension with: (a) To receive guidance for the use of ICTs in teaching practice. (b) Have a rigorous evaluation of the digital teaching competence itself. (c) Have information on how to continue developing digital skills among teachers and finally, (d) Have a frequent assessment of digital competence among teachers.

**Table 4:** Results of the hypothesis contrast of the variable ’Participation in ICT teaching innovation projects’ with the dimensions of the SQD.

Dimension	Item SQD	U Mann-Whitney	Z	p	r
**Modelled**	1	9568	3.117	.002	.20
	2	9766.5	3.462	.001	.22
	3	10198	4.22	.001	.27
	4	10520	4.779	.001	.30
**Reflection**	5	9897.5	3.714	.001	.23
	6	9486.5	2.963	.003	.19
	7	9805.5	3.502	.001	.22
	8	9515.5	2.992	.003	.19
**Instructional Design**	9	10576	4.883	.001	.31
	10	10336	4.492	.001	.28
	11	10856	5.383	.001	.34
	12	10594	4.925	.001	.31
**Collaboration**	13	10409	4.595	.001	.29
	14	9893	3.689	.001	.23
	15	9433.5	2.847	.004	.18
	16	10029.5	3.898	.001	.25
**Authentic Experiences**	17	10302.5	4.426	.001	.28
	18	10267	4.33	.001	.27
	19	9401.5	2.784	.001	.18
	20	9640	3.238	.001	.20
**Feedback**	21	10509	4.773	.001	.30
	22	9072.5	2.217	.027	.14
	23	9071.5	2.224	.026	.14
	24	8938	1.984	.047	.13

### Analysis of the relationships between ICT Coordination and the SQD

Finally, we respond to the second variable of the third objective, analyzing the relationships that exist between the characteristics of ICT teaching among staff and the performance of the position of ICT Coordinator in educational centers.

There are statistically significant differences in performance/non-performance in the role of ICT coordinator with 9 of the 15 variables of the six dimensions of the SQD (Table 5). (1) In the "Modelling" dimension with: (a) Observing many examples of ICT use in educational contexts. (b) Knowledge of ICT use in educational contexts to integrate, in an autonomous way, technological resources into professional practice. (c) Observe many examples of good ICT educational practices that inspire application in the classroom and (d) Receive specific demonstrations on the potential of ICT use in education. (2) In the "Instructional Design" dimension with: (a) To have received sufficient advice in the design of ICT-enriched activities and (b) have learned how to integrate ICT into the classroom teaching practice itself. (3) In the "authentic experiences" dimension with: (a) To have had many occasions to assess different ways of using ICTs in the classroom and (b) Have learned how to use ICT in the classroom through practices with trainers. (4) In the "Feedback" dimension with: (a) Get enough feedback on how to develop digital competition in the future. There were no significant differences with any of the variables of the "reflection" and "collaboration" dimensions of SQD.

**Table 5:** Results of the hypothesis contrast of the variables ’ICT coordination’ with the dimensions of the SQD.

Dimension	Item SQD	U Mann-Whitney	Z	p	r
**Modelled**	1	4.745	2.245	.025	.14
	2	4.960.5	2.787	.005	.18
	3	4.711.5	2.143	.032	.14
	4	4.768.5	2.355	.019	.15
**Reflection**	5	4.413	1.394	.163	.09
	6	4.531	1.692	.091	.11
	7	4.164.5	0.746	.455	.05
	8	4.087	0.551	.581	.03
**Instructional Design**	9	4.677.5	2.05	.040	.13
	10	4.845	2.496	.013	.16
	11	4.274.5	1.027	.304	.06
	12	4.390	1.323	.186	.08
**Collaboration**	13	4.466	1.516	.129	.10
	14	4.555	1.751	.080	.11
	15	3.680.5	-0.482	.630	.03
	16	4.356.5	1.232	.218	.08
**Authentic Experiences**	17	4.681	2.074	.038	.13
	18	4.734	2.193	.028	.14
	19	446.5	1.462	.144	.09
	20	3.978	0.276	.782	.02
**Feedback**	21	4.516	1.643	.100	.10
	22	4.547.5	1.733	.083	.11
	23	4.951.5	2.778	.005	.18
	24	4.548.5	1.743	.081	.11

## Discussions

The objectives of this study were, in the first place, to identify characteristics of teaching practices with digital technologies and digital teaching competence for the educational integration of ICTs, according to the perception of primary and secondary education teachers, through self-assessment tools that were applied just before the pandemic. A second objective was to know the methodological perspective present in the initial and ongoing training of teachers before the closing of schools due to the pandemic. Finally, we aimed to analyze the relationships between this training approach and participation in ICT-based educational innovation projects, on the one hand and the performance of the ICT Coordinator, on the other.

The theoretical and practical implications of this study derived from Objective 1 (O1) are as follows: (O1.1) The classroom was the most common educational space for the integration of digital technologies. (O1.2) There was moderate use of the virtual classroom, understood as the use of LMS platform (e.g. Moodle), classroom management (e.g. ClassDojo) or other web 2.0 resources (e.g. blogs). (O1.3) The b-learning modality was rare, as reflected by the results of the use of the "inverted classroom" or "flipped-classroom". (O1.4) Digital technologies were used more frequently by teachers as a resource to support expositional teaching practices (read/see/listen). (O1.5) Teaching methodologies enriched with less frequent technologies were: (i) collaborative activities; (ii) learning tasks aimed at the creation or production of digital resources by students; and (iii) ICT-supported communication activities. (O1.6) The most frequent learning outcomes that teachers achieved with the use of ICTs were related to "understanding", i.e., student achievement involving competencies for classification, explanation, or question formulation. "Synthesis" and "evaluation" are the least common learning outcomes. With regard to objective 2 (O2), the most relevant results were as follows: (O2.1) The teachers considered that they had not had sufficient opportunities to work, together with other colleagues, on the use of ICT in education, such as projects for didactic innovation or the realization of educational materials. (O2.2) The model of initial or permanent teacher training reveals a number of characteristics that could contribute to the understanding of the phenomenon experienced during "emergency remote education":(i) Although modeling has been revealed as an effective practice in teacher training [37], there is a lack of knowledge of real-world cases on ICT integration, insufficient teaching experience with ICT and little use of demonstration and observation of teaching practices enriched with digital technologies. (ii) Training has offered few opportunities for reflection on attitudes toward ICTs or communication of teaching experience itself, as well as on the role of technologies in education and its challenges. (iii) Technical and pedagogical advice for the use, design and development of technology-based practices and resources is considered insufficient.

Whatsmore, with regard to objective 3 (O3), the most outstanding results were: (O3.1) The existence of statistically significant differences in participation-non-participation in ICT innovation projects in all variables of the SQD. A relevant conclusion of this study is the relationship between the participation of teachers in ICT didactic innovation projects and the development of lifelong learning with greater opportunities for modeling, reflection, counselling, collaboration and knowledge of educational experiences. (O3.2) (O3.2) The existence of statistically significant differences in performance-non-performance of the ICT coordinator role with 9 variables belonging to the SQD’s "Modeling", "Instructional Design", "Authentic Experiences" and "Feedback" dimensions. It is concluded that the ICT coordinators show more favorable conditions toward a model of lifelong learning based on observation and demonstration, with greater external support and training to integrate technologies into teaching practice, supported by a direct knowledge of ICT use modalities in the classroom and with sufficient feedback to adequately develop their digital competence.

Recent advances in research on the SQD model support the results and proposals of this study in relation to teacher training [38]. In particular, the search for an integrated approach to the development of digital teaching skills concludes with the identification of three relevant strategies: (1) the use of the demonstration, through examples, by the models to be followed (to show quality practices); (2) the facilitation of the realization of ICT-enriched classroom educational practices (to gain experience); And (3) advice and support for the design of ICT educational activities (to stimulate and guide digital education plans). These three strategies are considered to be a "roadmap" of an inclusive model of competence development for the curriculum integration of digital technologies by teachers.

How it has been shown according to evidence, the educational integration of ICT’s reveals a number of weaknesses that have had an impact on the development of "emergency remote education": the lack of evaluation of the digital competence of the students, the lack of experience in the elaboration of teaching materials to support and which are appropriate to the individual differences or the limited use of online didactic communication. Both initial and ongoing teacher training does not achieve optimal results in relation to the development of this competency [39, 40]. In line with our results, various studies in the Spanish context conclude that teachers do not yet have sufficient level of digital competence [41, 42].

On the other hand, the model of initial or permanent teacher training (SQD) reveals a number of characteristics that could contribute to the understanding of the phenomenon experienced during «emergency remote education». Firstly, it is evident, although modelling has been revealed as an effective practice in teacher training [43], that there is a lack of knowledge of real cases on ICT integration, insufficient teaching experience with ICT and little use of demonstration and observation of teaching practices enriched with digital technologies. Secondly, training has offered few opportunities for reflection on attitudes toward ICTs or communication on teaching experience in itself, as well as on the role of technologies in education and its challenges. Training should address both beliefs and behaviors, be integrated into the teaching of subjects and placed environments of teachers and connect theory with practice and teachers should be trained through communities of practice and vocational learning [44–46]. Thirdly, it is evident that technical and pedagogical advice for the use, design and development of technology-based practices and resources is insufficient. A recent study found that only a third of teachers considered their school well prepared to use digital media before the closing of classrooms [47]. Fourthly, teachers do not sufficiently appreciate the relevance of cooperation, have had little chance of sharing experiences and mutual assistance is not frequent. It has been shown that collaborative work during teacher training in digital skills allows the acquisition of more knowledge and experience than individual work [48, 49]. Fifthly, teachers perceive a lack of motivation (incentives) toward the educational integration of ICTs and lack of time to assess new practices and receive mentoring on these initiatives. Teachers should have the opportunity to observe, reflect upon and experience how digital technologies can be used in teaching-learning activities [50]. In sixth place, there is evidence of the need to carry out, on a regular basis, an assessment of digital teaching competence that allows the design of training appropriate to the degree of development of knowledge, skills and attitudes of the teachers. The use of the strategies described in the SQD model has been shown to increase the practical levels of the TPACK [51] on the relationships between Curriculum Content (CK), Technology (TK) and Pedagogy (PK) of teachers in training [52]. Teachers need initial and ongoing training that is designed according to a series of principles that have been shown to be effective by educational research [53]: (1) orientation to curricular content; (2) use of active learning strategies; (3) involvement of teachers in collaboration; (4) use of models and/or modelling; (5) facilitation of coaching and expert support; (6) availability of time for feedback and reflection; and (7) sustained medium- and long-term duration. Voithofer & Nelson [54] investigated how teacher trainers implement the TPACK model [55], that is, the complex integration of curricular, pedagogical and technological knowledge into their training programs. The results of this study showed that the adoption of the TPACK was quite limited and consequently, an adequate training model for the integral development of digital teaching competence is not being applied. Since content, context and pedagogy are inseparable components of teacher knowledge [56], the educational integration of digital technologies is inseparable from the varied knowledge and practices applied by teachers. Therefore, training programs must go beyond exposure and reorient themselves toward an integrated experience that includes modeling and multiple opportunities for the use of technologies in specific educational contexts that allow for validation of instructional designs and digital resources. Alemdag et al. [57] designed and implemented a teacher training program based on the TPACK model. The results showed the need to identify the specific requirements of teachers, foster an active role from the teachers and provide opportunities to collaboratively design and develop digital educational resources. It is evident that there is a clear need to use the TPACK model with the integration of its three knowledge frameworks, as well as a greater contextualization of the use of technologies in the classroom. Collaboration among teachers is another recurring factor in literature.

An important conclusion of our study regards the relationship between the participation of teachers in ICT didactic innovation projects and the development of lifelong learning with greater opportunities for modelling, reflection, counselling, collaboration and knowledge of educational experiences. Positive effects have also been revealed with regards to the digital teaching competence of teachers who develop the role of ICT Coordinator in centres.

The results of this research are consistent with studies that highlight the importance of collaboration in teacher training, through different strategies of social practice. Spiteri et al. [58] conclude in a study on digital teacher competence in primary education teachers, that continuing teacher training should offer opportunities to apply classroom technologies collaboratively, encourage reflection on teaching practice with ICT and promote feedback among peers. Management teams should provide spaces and times for this communication between faculty members. It is concluded that mutual support among teachers facilitates the development of innovation with digital technologies. Rodriguez-Tiana et al. [59] studied teachers’ adoption of innovation by analyzing 40,235 shared learning designs across online professional communities within Graasp (https://graasp.eu/), a non-profit digital education platform used by more than 35,000 teachers around the world. The Knowledge Appropriation Model (KAM) [60] was used to identify different social practices and their relationship to the implementation of didactic innovations. This model identifies three categories of practices: (a) Knowledge maturation: Individual teacher creations are shared and transformed to be transferable to other educational contexts; (b) Knowledge scaffolding: Teachers request and receive help from other partners in the face of certain pedagogical problems arising in their professional practice; (c) Knowledge Appropriation: Explains how collectively developed knowledge is subsequently applied individually. The results show how the three practices have a strong relationship with the adoption of innovations concerning technologies in the classroom, concluding the relevance of social practices in the effective integration of technologies into teaching practices. Finally, Ley et al. [61] showed the benefits of an inventive Teacher Innovation Laboratory (TIL) program, based on the replacement of a linear model of knowledge transmission with a social dynamics approach in co-construction, co-creation, reflection, collaboration and knowledge integration between educational researchers and in-service teachers. The results support the importance of carrying out systematic and prolonged co-creation processes among researchers and teachers to facilitate the curricular integration of ICTs, through the adoption of methodologies appropriate to the context of digital education. It was concluded that the adoption of innovative practices by teachers requires teacher training that promotes social practices, where knowledge with sound scientific basis is co-constructed and which formalise educational proposals that are applied in real contexts where results can be evaluated.

With regard to the pedagogical approach to teacher training, there is an evident need for teachers to take a more active role and have opportunities to design and implement ICT educational practices in specific contexts. Martinez [62] identified three beneficial strategies in teacher training for ICT integration: (A) teacher collaboration, (b) digital pedagogy and (c) teacher needs. Collaboration, through open communication and feedback, contributes to meaningful learning and offers the opportunity to evaluate one’s teaching practices from the experience of other colleagues. Furthermore, training must lead to the development of didactic skills to critically assess the impact on learning in the use of digital tools. Finally, it is noted that training usually offers courses whose design does not include the individual and context characteristics of the teaching staff, decreasing the relevance of these learning experiences and their transfer to classroom practice. Fernandes et al. [63] through a review of the literature, identified three theoretical frameworks in ICT teacher training: (1) focus on the technological object: emphasis on the role of different tools and digital resources in teaching; (2) curriculum renewal: proposals of formative models for the use of digital technologies and pedagogical approaches based on inquiry; (3) cognitive processes: constructivist and conceptual knowledge-oriented approaches. It is evident that teachers do not clearly perceive the benefits of digital education in their professional development. It is also evident that most studies reveal that short-term courses or workshops are inadequate strategies to achieve change in teaching practices.

The online modality has been studied and there are similarities with our results regarding the propriety of adopting a collaborative, contextualized, practice-oriented and modeling approach. Bichler et al. [64] carried out the design, implementation and impact study of an online training course for secondary education teachers who were in transition to emergency remote education during the COVID-19 pandemic. The teacher training model used in this research presents a cycle that involves personalization, collaboration (teachers, researchers and ICT experts) and the viewing of the curriculum through the web. It is oriented toward the implementation of a constructivist pedagogy to customize didactic units on the web based on the results of the students. The results of this study show the value of context-specific online training in providing educational responses to specific student demands. Bragg et al. [65] analyzed, through a systematic literature review, the most effective practices for online teacher training and identified components of program design that improve curriculum and pedagogical knowledge. As a result of the study, it is confirmed that the most relevant elements are the promotion of participation and collaboration; the systematic use of guidance and scaffolding; the use of contextualized learning activities; the application of knowledge and skills acquired in practice; and flexible, objective-oriented design.

This research has some limitations. First, the sample of the study represents teachers from educational centers in a Spanish region who are characterized by having developed an initial digital education plan and who have participated in teacher innovation projects. Consequently, the transfer of results to all teachers should be considered with caution. Secondly, data obtained through self-report questionnaires reflect exclusively the teachers’ own perception and for a deeper analysis of the phenomenon, data collection would be required in classrooms and centers based on direct observations of teaching and organizational practices. Thirdly, this research will be placed within a very specific time frame which, unexpectedly for researchers, became a point of scientific interest, due to the consequences that the closure of educational institutions has had on the use of ICT for education.

With regard to future studies, we believe that it is necessary to redefine teacher training for the integration of ICT into more contextualized, reflective and participatory models. Consequently, it is essential to place the digital teaching competence within the framework of the Digital Education Project of the educational center and with this development in mind, we are beginning to apply a training model based on Design Thinking with very promising results.

This training perspective starts from the discovery of educational reality (initial diagnosis and definition of challenges) and continues with the interpretation of the context (identification of teaching practices and feasibility analysis) in a proactive manner by teachers. As a result, proposals for action are defined and priorities are established that are implemented for experimentation and where appropriate, reformulation, within a cyclical, iterative and continuous improvement process. In this model, the groups are made up of teachers from the same school and the trainers take on a role as dynamizers, guides and facilitators of the process.

The closure of schools has revealed a number of problems [1]. On the one hand, a pedagogical problem derived from the majority use of transmissive teaching, centered on the teacher, that does not fit with the innovative (disruptive) models of online education. On the other hand, it has been shown that teachers have insufficient training to carry out their guiding function with students outside the classroom. Moreover, these difficulties have generated an uncritical defense of face-to-face education, which does not consider its structural limitations for flexible and transformative education. Finally, it has been understood that the "new normal" consisted of a return to the educational status quo but this return does not seem desirable if the pandemic is seen as an opportunity for the transformation of the education system.

In conclusion, the development of digital competence is a fundamental component of the initial and ongoing training of teachers. However, it is a complex process that includes various strategies, for which there is not yet an integrated approach to improve teacher learning about the pedagogical uses of digital technologies. The most effective model for developing teacher digital competence is based on offering experiences that integrate digital technologies into learning as part of their training [66]. It is not enough to provide teachers with access to ICT; time to experiment and technical training on certain digital tools is also required. Reflective knowledge and skills needed to integrate digital technologies into teaching practice should be developed in teacher training programs [39].

## Supporting information

S1 Fig Phases of the research design. (TIF)

S1 File Survey PIED. (PDF)

S2 File Data survey PIED. (XLSX)
