# Supplementary figures and images for: The educational integration of digital technologies preCovid-19: Lessons for teacher education
Source: PLoS One. 2021 Aug 19;16(8):e0256283. doi: 10.1371/journal.pone.0256283 (PMC8375994; doi:10.1371/journal.pone.0256283)

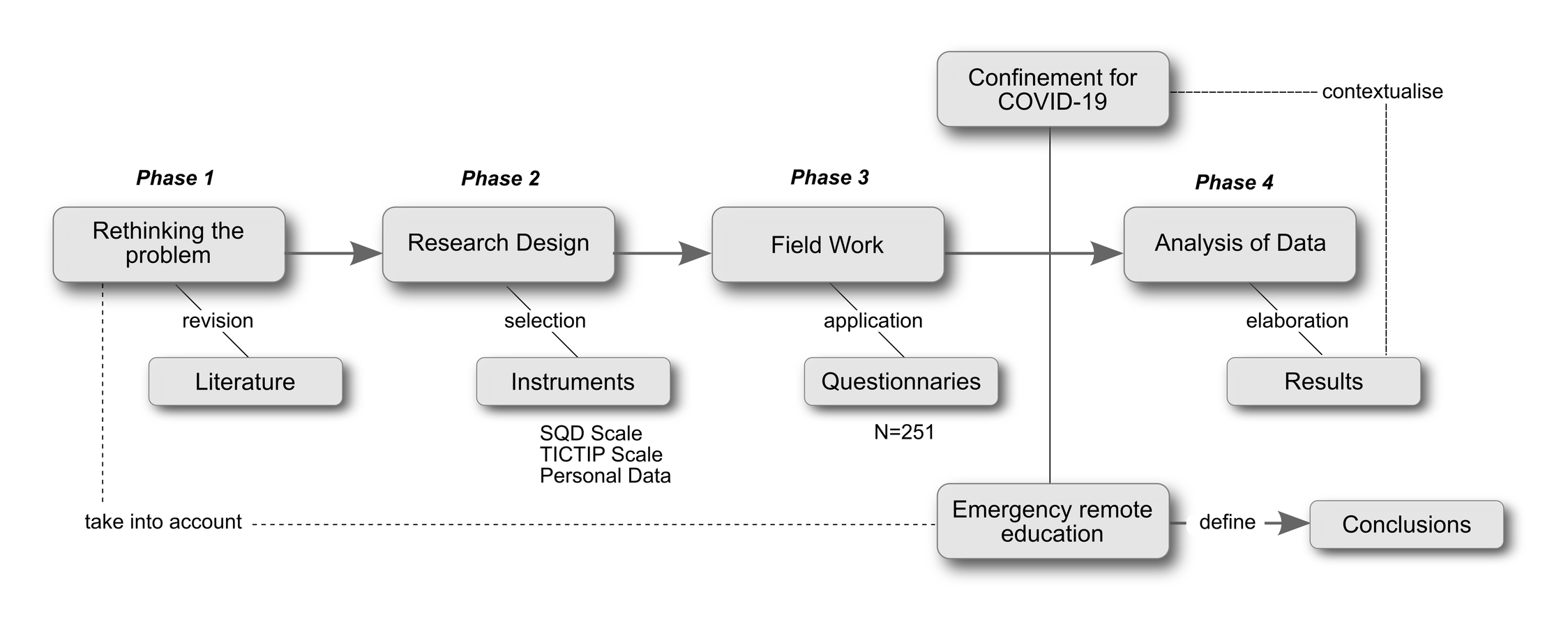

Supplement: S1 Fig — (TIF) [file pone.0256283.s001.tif]
