# Supplementary material for: The educational integration of digital technologies preCovid-19: Lessons for teacher education
Source: PLoS One. 2021 Aug 19;16(8):e0256283. doi: 10.1371/journal.pone.0256283 (PMC8375994; doi:10.1371/journal.pone.0256283)
Supplement: S1 File — (PDF) [file pone.0256283.s002.pdf]

«Plan Integral de Educación Digital (PIED) para la mejora de los resultados de aprendizaje en Educación Primaria y Secundaria». Ayudas destinadas a la realización de proyectos de investigación en los Centros públicos de I+D+i de la Comunidad Autónoma de Extremadura 2019-2022. Ref: IB18088

Descripción de la práctica docente habitual con TIC

1 = Nunca | 2 = Casi nunca | 3 = A veces |
4 = Con frecuencia | 5 = Muchísimas veces | 6 = Siempre

Espacios que utilizo para la enseñanza-aprendizaje con TIC	1	2	3	4	5	6
Aula-clase	○	○	○	○	○	○
Aula-tecnología / Aula-informática / Aula-ordenadores	○	○	○	○	○	○
Aula-laboratorio	○	○	○	○	○	○
Aula-Educación Física	○	○	○	○	○	○
Biblioteca	○	○	○	○	○	○
Espacio personal del estudiante (v.gr. hogar)	○	○	○	○	○	○
Aula invertida o Flipped Classroom (aula/s + espacio personal del estudiante)	○	○	○	○	○	○
Aula virtual (v.gr. ClassDojo, Moodle, Blogs, ...)	○	○	○	○	○	○
**Resultados de aprendizaje que espero obtener con el uso de las TIC**	**1**	**2**	**3**	**4**	**5**	**6**
Conocimiento (definir, identificar, recordar, enumerar, ...)	○	○	○	○	○	○
Comprensión (clasificar, explicar, preguntar, seleccionar, ...)	○	○	○	○	○	○
Aplicación (demostrar, encontrar, predecir, construir, ...)	○	○	○	○	○	○
Análisis (diferenciar, relacionar, comparar, ...)	○	○	○	○	○	○
Síntesis (generalizar, combinar, concluir, explicar razones para, ...)	○	○	○	○	○	○
Evaluación (criticar, dar argumentos a favor y en contra, juzgar, reflexionar, ...)	○	○	○	○	○	○
Actitudinal (mostrar conciencia hacia, ser receptivo a, valorar, ...)	○	○	○	○	○	○
Psicomotor (hacer, realizar, dibujar, desarrollar un ejercicio físico, ...)	○	○	○	○	○	○
**Tipo de práctica docente que realizo con TIC**	**1**	**2**	**3**	**4**	**5**	**6**
Leer/Ver/Escuchar (expositiva)	○	○	○	○	○	○
Colaborar (cooperativa)	○	○	○	○	○	○
Debatir-Reflexionar (comunicativa)	○	○	○	○	○	○
Investigar (indagativa)	○	○	○	○	○	○
Practicar (aplicativa)	○	○	○	○	○	○
Producir (creativa)	○	○	○	○	○	○

Por favor, compruebe que ha respondido a todos los ítems

Este cuestionario forma parte de una investigación del grupo «Nodo Educativo» de la UEx y está dirigida a profesorado de Educación Primaria y Secundaria. Su finalidad es conocer cómo ha sido la formación recibida sobre tecnología educativa e identificar los tipos de prácticas docentes con el uso de TIC. El cuestionario es anónimo y le llevará, aproximadamente, 10 minutos completarlo.

Le agradecemos el tiempo y dedicación empleados en realizar este cuestionario. Todos los datos recogidos serán tratados con total confidencialidad tanto en su análisis como en su divulgación y comunicación. La participación en este cuestionario implica el consentimiento informado en esta investigación. Cualquier participante tiene derecho a abandonar su realización cuando lo considere.

1 = Totalmente en desacuerdo | 2 = En desacuerdo | 3 = Ligeramente en desacuerdo |
4 = Ligeramente de acuerdo | 5 = De acuerdo | 6 = Totalmente de acuerdo

	Durante mi formación docente inicial y/o permanente...	1	2	3	4	5	6
1	He visto muchos ejemplos de uso de las TIC en contextos educativos.	○	○	○	○	○	○
2	He observado suficiente uso de las TIC en contextos educativos como para poder integrar por mí mismo estos recursos tecnológicos en mi práctica profesional.	○	○	○	○	○	○
3	He visto ejemplos de buenas prácticas educativas con TIC que me han inspirado para aplicarlas en mis aulas.	○	○	○	○	○	○
4	He recibido demostraciones concretas del potencial de uso de las TIC en la educación.	○	○	○	○	○	○
5	He tenido la oportunidad de reflexionar sobre el rol de las TIC en la educación.	○	○	○	○	○	○
6	He debatido sobre los desafíos de integrar las TIC en la educación.	○	○	○	○	○	○
7	Se me ha ofrecido la oportunidad de debatir sobre mi propia experiencia con las TIC en las aulas.	○	○	○	○	○	○
8	Hubo ocasiones específicas en las que se debatió sobre la actitud general hacia las TIC en la educación.	○	○	○	○	○	○
9	He recibido suficiente asesoramiento en el diseño de actividades de aprendizaje enriquecidas con TIC.	○	○	○	○	○	○
10	He aprendido cómo integrar TIC en mi práctica docente en el aula.	○	○	○	○	○	○
11	He recibido apoyo técnico y pedagógico para la elaboración de materiales didácticos.	○	○	○	○	○	○
12	He obtenido mucho apoyo para desarrollar actividades y proyectos educativos enriquecidos con TIC.	○	○	○	○	○	○
13	He tenido bastantes ocasiones en las que he trabajado junto a otros colegas sobre el uso de TIC en la educación (p.ej. proyectos de innovación didáctica, realización de materiales educativos, etc.).	○	○	○	○	○	○
14	Me he convencido de la importancia de la cooperación con relación al uso de las TIC en la educación.	○	○	○	○	○	○
15	Los docentes se ayudaron entre sí para el uso de las TIC en contextos educativos.	○	○	○	○	○	○
16	He compartido experiencias docentes sobre el uso de las TIC.	○	○	○	○	○	○
17	Hubo bastantes ocasiones en las que pude valorar diferentes formas de usar las TIC en el aula.	○	○	○	○	○	○
18	He sido capaz de aprender a usar TIC en las aulas a través de prácticas con formadores.	○	○	○	○	○	○
19	Se me motivó para que adquiriera experiencia en el uso de las TIC en las aulas.	○	○	○	○	○	○
20	Los propios docentes se motivaron entre ellos cuando intentaron usar las TIC en un contexto educativo.	○	○	○	○	○	○

Por favor, compruebe que ha respondido a todos los ítems

		1	2	3	4	5	6
21	He recibido suficiente asesoramiento sobre el uso de las TIC en mi práctica docente.	○	○	○	○	○	○
22	Mis competencias sobre el uso de las TIC han sido evaluadas a fondo.	○	○	○	○	○	○
23	He obtenido suficiente feedback (retroalimentación) sobre cómo desarrollar en el futuro mi competencia sobre el uso de las TIC.	○	○	○	○	○	○
24	Mis competencias en el uso de las TIC en el aula fueron evaluadas regularmente.	○	○	○	○	○	○

Escala sobre integración educativa de las TIC

1 = Nunca | 2 = Casi nunca | 3 = A veces |
4 = Con frecuencia | 5 = Muchísimas veces | 6 = Siempre

		1	2	3	4	5	6
1	Utilizo el ordenador para elaborar recursos de aula, material didáctico y actividades de evaluación.	○	○	○	○	○	○
2	Uso Internet, u otra tecnología de información, para la búsqueda de información que facilito a los estudiantes como recurso educativo complementario.	○	○	○	○	○	○
3	Dedico tiempo en la selección de medios o recursos TIC que se adecúan al currículo.	○	○	○	○	○	○
4	Utilizo software de presentación para mis clases expositivas.	○	○	○	○	○	○
5	Soy capaz de resolver problemas técnicos durante la clase (p.ej. el proyector/pizarra digital no reconoce el ordenador).	○	○	○	○	○	○
6	Aplico los objetivos, contenidos y criterios de evaluación relacionados con el desarrollo de la competencia digital que establece el currículo.	○	○	○	○	○	○
7	Utilizo el ordenador para registrar o editar sonidos/música como material didáctico.	○	○	○	○	○	○
8	Uso las TIC para introducir nuevas metodologías de enseñanza.	○	○	○	○	○	○
9	Utilizo el correo electrónico, la mensajería instantánea (v.gr. Rayuela) o la web para comunicarme con mis estudiantes.	○	○	○	○	○	○
10	Uso blog/web para compartir conocimientos o responder a cuestiones planteadas por los estudiantes.	○	○	○	○	○	○
11	Enseño a los estudiantes cómo buscar recursos útiles de la web para el aprendizaje académico.	○	○	○	○	○	○
12	En clases que uso TIC divido a los estudiantes en grupos.	○	○	○	○	○	○
13	Me aseguro que todos los estudiantes tienen suficientes recursos y habilidades TIC para realizar sus tareas académicas.	○	○	○	○	○	○
14	Facilito fichas de trabajo a los estudiantes cuando les pido que utilicen información web para completar sus deberes para casa.	○	○	○	○	○	○
15	Utilizo las TIC para fomentar la capacidad de pensamiento de alto nivel de los estudiantes, como la creatividad, el análisis y el juicio.	○	○	○	○	○	○
16	Evalúo la competencia digital de los estudiantes como complemento a la calificación académica basada en pruebas escritas (exámenes).	○	○	○	○	○	○
17	Valoro y califico el progreso en el aprendizaje de los estudiantes cuando participan en actividades en grupo con apoyo de TIC.	○	○	○	○	○	○

Por favor, compruebe que ha respondido a todos los ítems

		1	2	3	4	5	6
18	Evalúo mis prácticas docentes con TIC para mejorar mis clases.	○	○	○	○	○	○
19	Diseño tareas académicas con integración de TIC de modo que estudiantes sin ordenador en casa pudieran también participar.	○	○	○	○	○	○
20	Diseño diferentes actividades de aprendizaje de TIC para estudiantes con diferentes niveles de rendimiento.	○	○	○	○	○	○
21	Dedico tiempo a aprender y practicar competencias TIC.	○	○	○	○	○	○
22	Utilizo cursos y otros materiales online para mi formación profesional.	○	○	○	○	○	○
23	He asistido a congresos/jornadas o he leído revistas especializadas para aprender sobre métodos para la integración de las TIC.	○	○	○	○	○	○
24	Enseño a los estudiantes normas y reglas éticas sobre Internet antes que los estudiantes lo utilicen.	○	○	○	○	○	○
25	Exijo que los estudiantes respeten los derechos de propiedad intelectual.	○	○	○	○	○	○
26	Soy consciente de los problemas entre los adolescentes de adicción a Internet y el acceso a páginas de contenido adulto.	○	○	○	○	○	○
27	Comunico a los estudiantes cómo el uso abusivo de los dispositivos digitales puede afectar a su salud.	○	○	○	○	○	○
28	Hago seguimiento de los estudiantes con baja motivación y rendimiento académico por su adicción a Internet.	○	○	○	○	○	○

Datos de contexto

Centro Educativo ______

Sexo Mujer ○ Hombre ○

Edad (años) ______

Nivel educativo donde imparte docencia Educación Primaria ○ Educación Secundaria ○

Años de experiencia profesional ______

Situación laboral actual Interino/a ○ Funcionario/a ○

En caso de funcionario: Año de ingreso ______

He sido / Soy Coordinador/a TIC Sí ○ No ○

En caso afirmativo: Tiempo (número de meses) ______

En caso afirmativo: Actualmente con esta función Sí ○ No ○

He participado / participo en proyectos de innovación didáctica relacionados con las TIC Sí ○ No ○

En caso afirmativo: N.º de proyectos de innovación didáctica relacionados con las TIC ______

En caso afirmativo: Actualmente participo en un proyecto de innovación didáctica relacionado con las TIC Sí ○ No ○

Por favor, compruebe que ha respondido a todos los ítems
